# Advancing glioblastoma research by establishing a whole brain slice model

**DOI:** 10.3389/fonc.2026.1814381

**Published:** 2026-06-24

**Authors:** Katharina Fuchs, Clara Keller, Bianca Layer, Katja Nadler, Ellaine Salvador, Tobias Weigel, Bastian Christ, Sofia Dembski, Ralf-Ingo Ernestus, Mario Löhr, Carsten Hagemann

**Affiliations:** 1 Division Experimental Neurosurgery, Department of Neurosurgery, University Hospital Würzburg, Würzburg, Germany; 2 Translational Center Regenerative Therapies TLC-RT, Fraunhofer Institute for Silicate Research ISC, Würzburg, Germany

**Keywords:** AlamarBlue assay, brain tumor, *ex vivo* modeling, glioblastoma, life-death staining, organotypic brain slice cultures, spheroids

## Abstract

Organotypic brain slice cultures are well-established and valuable three-dimensional (3D) models in neuroscience, particularly for cancer research. However, many existing 3D models rely on adult animals or are restricted to specific brain regions, such as the hippocampus. To address these limitations, we established a whole, large-format brain slice model that preserves the complex cellular architecture of the brain and its diverse cell populations. In addition, the use of 6-9-day-old postnatal mice allows a reduction in animal numbers by sectioning each brain into multiple contiguous slices of 300 µm thickness. The slices were maintained *in vitro* for 7–14 days under stable conditions and served as carrier tissue for brain tumor cell spheroids, thereby creating an *ex vivo* glioblastoma (GBM) model. To simulate tumor growth and invasion, spheroids of rat 9L/lacZ GBM cells were seeded onto the murine slices. Two different fluorescence-based methods were used to visualize and quantify the viability of the model. The AlamarBlue assay was used to identify the optimal culture medium and to longitudinally monitor whole-slice viability over a 10-day period. In parallel, the structural integrity of the brain slices and tumor invasion patterns were visualized using live/dead staining for up to 14 days post-slicing. Both methods demonstrated that the slices remained viable and stable for one week before a gradual decrease in viability. When co-cultured with GBM spheroids, the slices’ viability slightly decreased, likely due to the tumor’s invasive nature, which compromised the complex brain structures and cell networks. In summary, our *ex vivo* whole-brain slice GBM model preserves the complex organotypic brain and tumor structures essential for a robust *in vitro* GBM 3D model. The model stability for a minimum of 7 days, with the potential for extended culture duration, makes it a valuable tool in brain cancer research.

## 1 Introduction

Glioblastoma (GBM) is the most aggressive and treatment-resistant primary brain tumor in adults, characterized by rapid proliferation, diffuse infiltration, and profound intratumoral heterogeneity (1, 2). A defining feature of GBM is its dynamic interaction with the tumor microenvironment (TME), which comprises neurons, glial cells, immune populations, vasculature, and extracellular matrix components (3, 4). These interactions critically influence tumor progression, therapeutic resistance, and immune evasion (5–7). Traditional *in vitro* two-dimensional (2D) models, while invaluable, often fail to fully recapitulate the complexity of the human tissue and TME (8, 9). On the other hand, animals are widely used *in vivo* models, providing a whole-organism interaction network for pharmacokinetic studies, drug development and toxicity studies (10), but are constrained by ethical considerations (10–12). Considering the 3R principle – replacement, refinement and reduction – there is a growing demand for alternative models that reduce animal usage while maintaining physiological relevance (12, 13). In recent years, 3D *ex vivo* models have emerged as valuable tools for investigating tumor biology, behavior, and drug testing (14–19). The discrepancy between drug effects observed in 2D *in vitro* systems and those seen in preclinical or clinical studies underscores the need for improved models (14). One goal to enhance these systems is to mimic the TME and organotypic extracellular matrix to provide an adequate tumor-native tissue interaction (8, 20, 21). Organotypic brain slice cultures, derived from whole murine brains offer a promising *ex vivo* platform (15–18). These models preserve native tissue architecture and cellular diversity, enabling the study of tumor invasion, cell migration and therapeutic response in a controlled yet biologically authentic setting (8, 19, 22–24). By integrating tumor spheroids into intact brain slices, researchers can investigate GBM-TME interactions with high spatial and temporal resolution, while adhering to ethical standards and minimizing animal distress and number (7, 8). Such organotypic approaches are increasingly recognized as indispensable tools in GBM research, bridging the gap between reduction of *in vitro* systems and complex *in vivo* models (19). To address current challenges of a solid GBM model, we established a whole murine brain slice model that can be cultured for 7–14 days *in vitro*. This model allows for the detailed examination of tumor invasion and migration mechanisms by adding tumor spheroids onto the slices. Specific regions of the brain, such as the cortex, forebrain, midbrain, cerebrum, and hippocampus, can be targeted in this GBM model to gain a deeper understanding of the disease (25, 26). In this study, we employed two different fluorescence-based analysis methods to assess model health qualitatively (live/dead staining, LDS) and measure tissue viability quantitatively (AlamarBlue assay) (26–28). In addition, we visualized some selected molecular markers for proliferation (Ki67), invasion (matrix metalloproteinases (MMPs) -2 and -19) and neuronal activation of host tissue (c-FOS) by immunofluorescence microscopy. This model is intended to bridge the gap between conventional two-dimensional (2D) culture systems and complex *in vivo* models, providing a more physiologically relevant platform for investigating GBM biology and evaluating potential therapeutic approaches.

## 2 Materials and equipment

### 2.1 Whole brain slice preparation

- Laminar flow hood
- Phosphate buffered saline (PBS) (Sigma-Aldrich, Cat. No. D8537-500ML)
- Preparation medium: Minimal Essential Medium (MEM) (Gibco™, Cat. No. 32360026) supplemented with 1% GlutaMAX™ (Gibco™, Cat. No. 35050061), 1% v/v of a 45% v/v glucose solution (Sigma-Aldrich, Cat. No. G8769-100ML), 100 U/mL penicillin and 0.1 mg/mL streptomycin (Gibco™, Cat. No. 15140-122)

Note: Prepare this medium freshly for each preparation.

- Culture medium: MEM supplemented with 1% GlutaMAX™, 1% v/v of a 45% w/v glucose solution, 25% v/v normal horse serum (NHS) (Gibco™, Cat. No. 26050088), 25% v/v Hanks’ balanced salt solution (HBSS) (Gibco™, Cat. No. 14025050), 0.08% w/v L-Ascorbic acid (Sigma-Aldrich, Cat.No. A4544), 100 U/mL penicillin and 0.1 mg/mL streptomycin

Note: Store the culture medium at 4 °C during the culture time. Preheat to 37 °C before use. Even when stored at 4 °C, do not use this medium for longer than one week due to the low durability of L-ascorbic acid. Best way is to prepare multiple aliquots to add freshly to the medium on day of use. Keep it in the dark and light-protected.

- Water bath set to 37 °C
- CD1/Crl mice, other wild type or genetically modified mouse strains, postnatal 6–8 days.

Note: Organ removal was performed in accordance with the Policy of Ethics and the Policy on the Use of Animals in Neuroscience Research as approved by the European Communities Council Directive 2010/63/EU of the European Parliament and of the Council of the European Union on the protection of animals used for scientific purposes and the responsible local animal welfare authorities.

- Tweezers (surgical and tissue), scalpels and very sharp scissors
- Cell culture dishes (6 cm) and filter paper
- Vibratome (e.g. Compresstome^®^ vibratome)

Note: We are using the Compresstome^®^ VF-300 (Precicionary Instruments LLC). However, any other vibrating microtome is suitable, if a cutting thickness of 300 µm and the other settings are adjustable.

**Figure 1:**
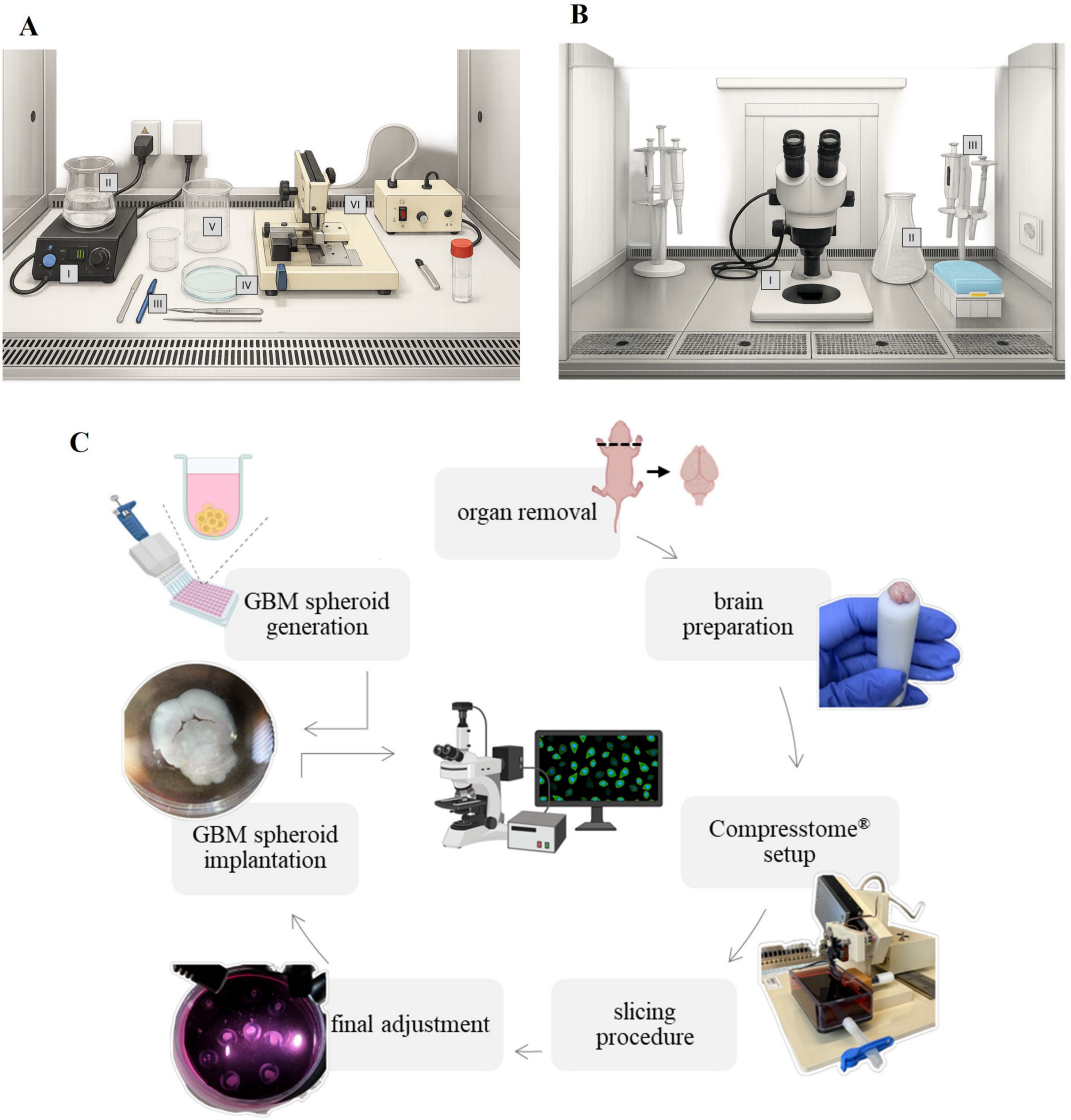
Step-by-step instruction of the slicing procedure for whole brain slice preparation. **(A)** Setup of brain slice preparation under sterile conditions. A heated stirring plate (I) provides continuous stirring of freshly prepared agarose (II). Sterilized tools, including forceps, scalpels and spatulas, are arranged for easy access (III). A petri dish (IV) and a waste container (V) are positioned next to the Compresstome^®^ (VI). Figure created with illustrae.co. **(B)** A stereo microscope placed inside the laminar flow hood assists in the carful removal of agarose from brain slices (I). A waste container (II) and pipettes with sterile tips (III) are used to handle excess medium. Figure created with illustrae.co. **(C)** Workflow of *ex vivo* brain slice generation and tumor implantation. GBM spheroids are generated at least 3 days prior to slicing in a BIOFLOAT™ 96-well plate. On preparation day, brains from postnatal day 6–9 mice are harvested, embedded in pre-warmed agarose, and sectioned into 300 µm slices using a Compresstome**^®^**. After removing agarose, slices are transferred onto membrane inserts in culture medium. One or two GBM spheroids are placed onto the slice surface per hemisphere and cultured at 37 °C, 5**%** CO_2_, and 95**%** humidity. Figure partly created with BioRender.

- Spatula with a flat and wide surface
- Histoacryl (B. Braun SE, Cat. No. 1050052) or other tissue adhesive
- Pasteur pipettes
- Stereomicroscope (e.g. Leica S9D) (**Figure 1**)
- Razor blades (Wilkinson Sword, Cat. No. 7005115E)
- Superglue (mfi Metall + Fastening Industrie, Cat. No. 71024)
- 6-well cell culture plate, flat bottom with lid
- Agarose Tablets ROTI^®^ Garose 0.5 g (Carl Roth, Cat. No. HP67.7)
- Inserts for 6-well cell culture plates with 0.4 µm transparent PET membrane

### 2.2 General cell culture and generation of spheroids

- 50 mL tubes and 1.5 mL Eppendorf microtubes
- Accutase^®^ Enzyme Cell Detachment Medium (Life Technologies, USA)
- BIOLFOAT™ 96-well cell culture plate (Sarstedt, Cat. No. 83.3925.400)

Note: It is necessary to use a low attachment well plate with round bottom shaped wells to achieve optimal spheroid formation and growth. Otherwise, the cells would attach to the surface of the well. We use the BIOFLOAT™ 96-well plate from Sarstedt AG, which is already pre-coated, round-shaped and ready-to-use.

- Carboxyfluorescein diacetate (CFDA)
- Note: We recommend using the Cell Trace™ CFSE Cell Proliferation Kit (Invitrogen™, Cat. No. C34554), which has been used for the example experiments described below. However, any other suitable kit based on CFDA from other manufacturers can be used.

- Cell specific culture medium for 9L/lacZ: Dulbecco’s Modified Eagle’s Medium (DMEM) containing 4.5 g/L glucose, sodium pyruvate, 3.7 g/L NaHCO_3_ and L-glutamine (Gibco™, Cat. No. 31885023) supplemented with 10% v/v heat-inactivated fetal bovine serum (FBS) (Gibco™, Cat. No. A5256701), 100 U/mL penicillin, and 0.1 mg/mL streptomycin.
- Cell specific culture medium for U87: Dulbecco’s Modified Eagle’s Medium (DMEM) containing 4.5 g/L glucose, sodium pyruvate, 3.7 g/L NaHCO_3_ and L-glutamine supplemented with 10% v/v heat-inactivated FBS, 2 x nonessential amino acids (NEA) (100 x stock, add 10 mL to 500 mL DMEM) (Gibco™, Cat. No. 11140050), 100 U/mL penicillin, and 0.1 mg/mL streptomycin.

Note: The composition of the cell culture medium was optimized for the specific cell line used in this study. Other cell lines may require alternative medium formulations, supplementation, or culture conditions to ensure appropriate viability and growth pattern within the brain slice model.

- Glass pipettes with pipetting aid.
- PBS without Ca^2+^ and Mg^2+^, low endotoxin
- Scepter™ 3.0 Handheld automated Cell Counter with 60 µm sensors (Merck Millipore, Cat. No. PHCC360050) or any other suitable cell counting device

Note: We are using the Scepter™ 3.0 Handheld automated Cell Counter with 60 µm sensors for the determination of the cell concentration. However, any other cell counting device is suitable. We recommend using the same system for all experiments.

- Vent cap canted neck cell culture flasks (75 cm^2^) (e.g. Sarstedt, Cat. No. 83.3911.002)

### 2.3 AlamarBlue™ assay

- AlamarBlue™ HS Cell Viability Reagent (Invitrogen, Cat. No. DAL1100).
- Cell line-specific culture medium
- Microplate-Reader suitable for 6-well plates and fluorescence detection (e.g. Tecan infinite 200 PRO^®^, Tecan Group)

### 2.4 Life-death-staining

- 15 mL tubes
- 6-well cell culture plate, flat bottom with lid
- Fluorescence microscope

Note: We used the Carl Zeiss Axiovert 40 CFL+Axio Imager M2 system for 2D tile microscopy.

- GelRed working solution: GelRed Nucleic Acid Gel Stain (10,000 × in water) (Biotum, Cat. No. 130 41003) diluted in water to a 30× working solution
- LDS-Staining-Solution: 1:10 Gel Red working solution, 1:100 Fluorescein diacetate (FDA, 5 mg/mL in Dimethylsulfoxid (DMSO)) (Sigma-Aldrich, Cat. No. F7378-5G), 1:10,000 Hoechst 33324 (2’-[4–133 Ethoxyphenyl]-5-[4-methyl-1-piperazinyl]-2,5’-bi-1H-benzimidazol trihydrochloride) (Thermo Scientific, Cat. No. 62249) dissolved in PBS
- PBS without Ca^2+^ and Mg^2+^, low endotoxin
- Aluminum foil

### 2.5 Fixing and paraffin-embedding of the slices

- 2% Agarose in PBS
- 4% Paraformaldehyde (PFA) in PBS (Sigma-Aldrich, Cat. No. 104.003)
- Dehydration station (Leica, HistoCore PEARL)
- Embedding cassettes (Resolab, Cat. No. 140-002)
- Filter paper
- Paraffin station (MEDITE Medical, TES99)
- PBS without Ca^2+^ and Mg^2+^, low endotoxin

### 2.6 Hematoxylin and eosin staining

- Eosin solution (Carl Roth, Cat. No. 3137.1)
- Ethanol (Carl Roth, Cat. No. K928.4)
- Hematoxylin solution (Carl Roth, Cat. No. T865.3)
- Xylene (VWR International, Cat. No. 28975360)
- Xylene containing mounting medium (Pertex™, MEDITE Medical, Cat. No. 41-4012-00)

### 2.7 Immunofluorescence staining

- Antibodies directed against c-FOS (Calbiochem, Cat. No. PC40), Ki67 (abcam, Cat. No. AB16667), MMP-2 (R&D, Cat. No. MAB902), and MMP-19 (Sigma-Aldrich, Cat. No. M-5309)
- Antibody dilution buffer (DCS Innovative Diagnostik-Systeme, Cat. No. AL120R500)
- Citric acid monohydrate (Sigma-Aldrich, Cat. No. 33114-500G)
- Hydrochloride acid (Sigma Aldrich, Cat. No. 1.00316.2511_P)
- Mounting Medium with DAPI (Abcam, AB104139)
- Normal Goat Serum (10%) (Invitrogen, Cat. No. 50062Z)
- Orbital shaker (Heidolph Scientific Products, Cat. No. 544.41200.00 0)
- PapPen (Science Services, Cat. No. N71310-N)
- Pressure Cooker (Amazon Basics, Cat. No. B093L4X2FW (ASIN))
- Secondary antibody AlexaFluor488 Goat anti-rabbit antibody (Invitrogen, Cat. No. A32723)
- Sodium chloride (Carl Roth, Cat. No. 0601.1)
- Tris for analytics (ITW Reagents, Cat. No. A1086,1000)
- Triton X 100 (Carl Roth, Cat. No. 3051.3)
- Tween20 (Carl Roth, Cat. No. 9127.2)
- Wet chamber (self-made)

## 3 Methods

### 3.1 Culturing and seeding of spheroids

1. Maintain cell lines of interest in 75 cm^2^ cell culture flasks, containing 12 mL of cell culture medium, at 37 °C, 5% CO_2_ and 100% humidity. Passage cells at sub-confluency (~60 – 80%) using Accutase to detach them from the culture flask.
2. Discard the medium, wash cells quickly with PBS and add 2.5 mL Accutase.
3. Incubate cells for about 10 min at room temperature. Check cell dissociation microscopically. When cells are properly detached, add cell medium containing FBS to stop the Accutase reaction.
4. Count cells with the Scepter™ 3.0 or any other suitable device.
5. Transfer calculated cell suspension into a 50 mL tube and add cell-specific medium to reach desired concentration. For 9L/lacZ spheroids we recommend 500 cells in 100 µL cell specific medium per well.
6. Seed cells in a 96-well BIOFLOAT™ plate to generate single spheroids in each well.
Note: It is necessary to use a low attachment well plate with round bottom shaped wells to achieve optimal spheroid formation and growth. Otherwise, the cells would attach to the surface of the well and form a 2D-cell layer. We use the BIOFLOAT™ 96-well plate from Sarstedt, which is already pre-coated, round-shaped and ready-to-use.
7. Pick spheroids on slice preparation day to perform either CFDA labeling or seed them directly onto the surface of the whole brain slices. Set up a 100 µL pipette to a low volume (10-30 µL) to reduce the amount of cell medium on the slice.
Note: We recommend growing the spheroids without any disturbance for at least three days until they can be picked for assays or seeding.

### 3.2 Set-up of the vibratome

1. Dissolve two agarose tablets in 50 µL PBS by heating up in a microwave. Place it with a stirring rod on a pre-heated stirring plate (50-60 °C) under the laminar flow hood (**Figure 1**). Ensure maintenance of set temperature to prevent agarose coagulation.
2. Position the vibratome under the laminar flow hood as well. Clean and thoroughly disinfect it to have maximum sterility (**Figure 1**).
3. Superglue a razor blade on its holder and place it inside the vibratome.
4. Set slice thickness to 300 µm. For our vibratome we use the following settings oscillation to 6 and advance to 3.5.

### 3.3 Preparation of the whole brain slices

1. Freshly prepare 200 mL preparation medium as outlined in 2.1. Store it at 4 °C and keep on ice until the preparation procedure starts.
Note: We recommend 200 mL for 3–4 mice.
2. Prepare 100 mL culture medium as outlined in 2.1 and prewarm it in a water bath to 37 °C.
3. Decapitate a mouse. Discard the body and keep the head on a petri dish with filter paper. All the following steps should be performed under the laminar flow hood to ensure sterile conditions.
Note: Make sure the mice are healthy. It will affect the quality of the whole brain slices immensely.
4. Remove the skin from the skull using a tweezer.
Note: For a better grip fix the mouth of the mouse with a big tweezer while removing the skin with a second tweezer.
5. Make a caudal T-section, open the skull and remove the bone and cartilage tissue with a surgical tweezer. Work very carefully to not damage the brain.
6. Remove the brain from the skull with a sterile small spoon or surgical spatula.
7. Apply histoacryl tissue glue onto the tissue holder of the vibratome and attach the brain with its ventral side to it, and the dorsal side on top.
8. Place the brain on the holder through the metal cylinder and pour agarose around it with a plastic Pasteur pipette to fix the brain tissue inside the cylinder.
9. To harden the agarose, use a clamp, which is pre-cooled to -80 °C. Hold the cylinder with the tweezers not longer than 1 min to avoid brain damage. Place the cylinder inside the vibratome.
10. Fill the vibratome tub with pre-cooled preparation medium to cover the whole cylinder.
11. Start the slicing procedure and take out the first sections as they consist only of agarose. As soon as there is brain visible, collect the slices in a glass petri dish, filled with pre-cooled preparation medium. We recommend using a spatula.
Note: The first slices can be done quickly and a bit thicker. After 3–4 cuts one must be more careful and cut the slices slowly and in the right thickness. If the blade runs too fast over the brain, the slices may rupture. The exact settings of the microtome may differ from different manufacturers and therefore must be optimized in preliminary experiments.
12. Work with a stereomicroscope to cut off the agarose from the slices with a scalpel.
Note: Make sure to not cut into the brain tissue as this causes tissue damage and reduces slice viability. To save the precious organ-derived models we recommend using the damaged slices directly for live/dead staining or other immediate analysis. Since they will not be cultured, the minimal damage will not affect live/dead staining results at this time point. Therefore, instead of discarding they are saved and utilized to deliver significant information for your research.
13. Fill a 6-well plate with 1 mL of culture medium per well. Prepare one well for each slice.
Note: The medium required is approximately 1 mL per well but will vary depending on the manufacturer of plates and inserts.
14. Place an insert, suitable for a 6-well plate, inside each well. Avoid air bubbles under the membrane to have maximum nutrient supply for the whole brain slice.
15. With a small spoon put some preparation medium inside the insert, then place a slice into it. Gently shake the plate to allow the slice to expand in the center of the insert. Remove the preparation medium with a 1000 µL pipette.
Note: Make sure that there is no medium on top of the slices. Otherwise, they will disintegrate. Nutrient absorption from the medium in the well takes place via the porous membrane of the insert.
16. Incubate the slices at 37 °C in a humidified atmosphere with 5% CO_2_.
17. Change medium daily for the first week. Afterwards, changing medium every other day is sufficient.

#### 3.3.1 Optional: fluorescence labeling of spheroids with CellTrace™ CFSE cell proliferation kit

18. Labeling the spheroids can help to localize them, measure size alteration, and analyze cell behavior. For this purpose, we utilize the CellTrace™ Cell Proliferation Kit.
Note: CFDA passively diffuses into the cells, reacting with intracellular compounds forming fluorescent conjugates, which are inherited by daughter cells. Thus, after CFDA removal, the fluorescence intensity will roughly be halved with each cell division. If you would like to have a stabilized fluorescence intensity over a longer period we recommend using transduced cells, which express green fluorescent protein or firefly luciferase.
19. Prepare a 10 mM stock solution with DMSO following the manufacturer’s instruction.
20. Freshly prepare the staining solution on the slicing and seeding day. Dilute the stock solution 1:1000 with pre-warmed PBS (37 °C) and add 500 µL per well in a 12-well plate.
21. Pick spheroids with a 100 µL pipette and incubate them for 45 min at 37 °C. We recommend staining 1–4 spheroids per well to avoid them to merge.
22. Wash spheroids for 30 min in pre-warmed cell specific medium. After that, the spheroids can be used to be seeded onto the slices.
23. Pick one spheroid and place it gently on top of a slice, one on each hemisphere. We use 1–2 spheroids per slice.
Note: The viability of the whole brain slices over the incubation time depends on the used tumor cell lines. The optimal handling and incubation time needs to be established before the experiments.

#### 3.3.2 Optional: picking unstained spheroids

24. Pick unstained spheroids directly from the BIOFLOAT^®^ plate as described above. Use 1–2 spheroids per slice.

### 3.4 AlamarBlue™ assay

1. Work under the laminar flow hood.
2. Dilute the AlamarBlue reagent 1:10 in cultivation medium.
3. Use sterile forceps to remove the insert from the well plate. Hold it in the air under the hood to avoid any contamination.
4. Use a 1000 µL pipette to discard the old medium and replace it with 1 mL AlamarBlue medium.
Note: The medium required is approximately 1 mL per well but will vary depending on the plates and inserts used.
5. Gently place the insert back to the well by avoiding air bubbles.
6. Incubate the slices for 24 h at 37 °C.
7. Read out fluorescence signal at plate reader with following parameters:
  • Excitation: 560 nm
  • Emission: 590 nm
  • Gain: optimize on first measurement, then use this value manually for all succeeding measurements

Note: To avoid contamination, keep the lid on the plate during the measurement. Initiate the first AlamarBlue measurement on day 0 and normalize the viability values of subsequent measurements to slice culture day 1 as base-line. The assay requires a 24 h incubation time. The assay reagent is non-toxic and can be applied to the same slice for multiple measurements. Therefore, remove the AlamarBlue medium after each measurement and replace it by fresh AlamarBlue medium for a follow-up measurement of the same slice 24 h later.

### 3.5 Live/dead staining

1. Prepare aluminum foil to cover samples in well plates.
2. Take a new 6-well plate.
3. Use forceps to transfer inserts containing whole brain slice cultures into 6-well plate.
4. Wash carefully with 500 µL PBS per well.
Note: As the slices can be damaged easily, it is advisable to pipette the PBS dropwise into the inserts. Do not drop the fluids directly onto the slices. After pipetting gently shake the plate until the slices are completely covered to make sure they stay intact.
5. From now on, protect samples from bright light.
6. Prepare the staining solution in a black tube or wrap aluminum foil around a regular one.
7. Add 500 µL of the staining solution per well dropwise.
Note: As the slices can be damaged easily, it is advisable to pipette the staining solution dropwise into the inserts. Do not drop the fluids directly onto the slices. After pipetting gently shake the plate manually until the slices are completely covered to make sure they stay intact.
8. Cover the well plate with aluminum foil and incubate samples for 10 min at room temperature.
9. Discard the staining solution.
10. Wash the whole brain slices with 1 mL PBS for 10 min. Remove the PBS carefully.
11. Add 1 mL PBS and leave slices in PBS until examination under a fluorescence microscope with filters for FDA with Excitation BP480/40 and Emission BP527/30 and for GelRed with Excitation BP545/25 and Emission BP605/70.
Note: Use the correct filters and microscope settings. Living cells appear green and dead cells red due to dye permeation through their membranes. Keep in mind that slices cannot be used for other staining or further evaluations after the live/dead staining as the dyes are toxic and permanent. Therefore, do not use all, but only some of the generated slices for evaluation of the live/dead ratio, unless it is your main experiment.

### 3.6 Fixing and paraffin embedding of the slices

1. Remove the culture medium from wells. Wash once with 500 µL PBS per well. Make sure to not pipette directly onto the slices as they may disrupt (see note from 3.5).
2. Fix slices in 4% PFA. Ensure the slice is surrounded by the PFA solution (both the apical and basal sides of the insert). Incubate for 2 h.
3. Wash again with PBS. Keep the slices in PBS until paraffin embedding.
4. Cut out the fixed slice from the insert but keep it on the membrane to avoid damage.
5. Drop 2% Agarose solution around the slice lying on the membrane to cover it completely. The agarose solution supports during the embedding process as the filter paper can attach to the slice and can destroy it.
6. Transfer the agarose-covered slice onto the embedding cassette lined with filter paper.
7. Initiate a dehydration process by immersing the cassette in the following solutions: 70% ethanol for 135 min, 80% ethanol for 45 min, 100% ethanol for 180 min, xylene for 90 min, paraffin for 120 min.
Note: Incubation times or ascending ethanol series may need to be adjusted and extended depending on the paraffin station employed.
8. Remove filter paper carefully as well as the lid of the cassette. Perform paraffin-embedding at paraffin station.
9. Store embedded samples at room temperature at a dry place.
10. Slice embedded samples at 2.5 µm thickness minimum one day before HE staining.

### 3.7 Hematoxylin and eosin staining

1. Deparaffinate and rehydrate slices by following descending ethanol series: xylene 5 min, xylene 5 min, 96% ethanol 3–5 dips, 96% ethanol 3–5 dips, 70% ethanol 3–5 dips, 50% ethanol 3–5 dips, deionized water.
Note: Sway until turbulences clear to have a fully rehydrated slice. Increase time if necessary.
2. Put slices in hematoxylin for 6 min at room temperature to stain cell nuclei.
3. Wash out dye with deionized water, followed by bluing with tap water for 5 min.
Note: Keep water running in a low rate, avoid dropping it directly onto slices.
4. To stain cytoplasm and extracellular matrix, put slices in eosin for 6 min at room temperature.
5. Wash out dye with deionized water. Rinse until water runs clear.
6. Mount slices with aqueous mounting medium or perform ascending ethanol series to mount with xylene mounting medium.

### 3.8 Immunofluorescence staining

1. Prepare a 20 mM citrate buffer (pH 6.0) by dissolving 8.4 g citric acid monohydrate and 4.2 g sodium chloride in 2000 mL deionized water. Adjust the pH to 6.0. Dilute the buffer 1:1 with deionized water to obtain a final concentration of 10 mM, and add Tween20 to a final concentration of 0.005%.
2. Prepare 10× TBS-T by adding 121.1 g Tris, 169.4 g sodium chloride, 100 mL hydrochloride acid and 2 mL Triton X 100 to 1900 mL deionized water. Dilute the solution 1:10 and add 0.01% Tween20 to gain 1× washing buffer.
3. Boil slices for 20 min in 1.5 L of 10 mM citric buffer in a pressure cooker. After that cool down for 20 min.
4. Edge the samples on the slide with a PapPen.
5. Wash two times in 1× TBS-T for 5 min each on an orbital shaker.
6. Place slices in a wet chamber and pipette 50 µL of blocking solution (10% goat serum) onto each sample. Incubate for 30 min at room temperature.
7. Discard blocking solution and add 50 µL of primary antibodies pre-diluted with antibody dilution buffer (Ki67 1:1000, MMP-2 1:100, MMP-19 1:100, c-FOS 1:500) and incubate overnight at 4 °C in a wet chamber.
8. Discard the antibody solutions and wash slices two times in 1× TBS-T for 5 min each.
9. Add secondary antibody diluted 1:1000 in 50 µL antibody dilution buffer per sample for 1 h at room temperature and in the dark.
Note. Use secondary fluorochrome-labeled antibodies according to your species system. Thus, we used AlexaFluor488 Goat anti-rabbit antibody.
10. Discard antibody solutions and wash twice in 1× TBS-T for 5 min each.
11. Mount slices with mounting medium containing DAPI and store samples at 4 °C until microscopy.

## 4 Results

**Figure 2:**
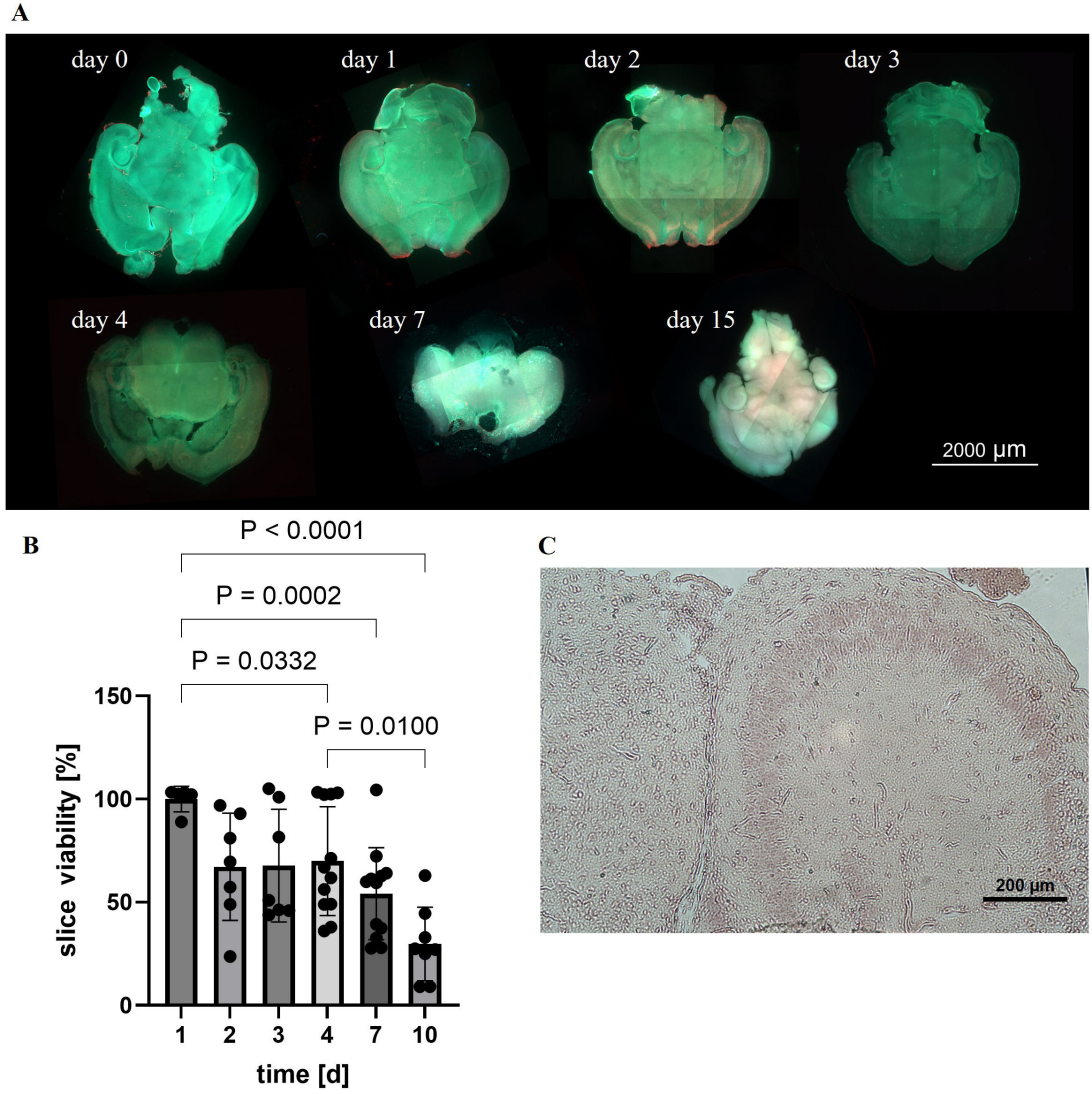
Establishment and maintenance of the *ex vivo* whole brain slice model. **(A)** Representative fluorescent images of LDS stained whole brain slices over time (day 0-15). Slices remained stable and viable up to 15 days, demonstrating long-term culture ability. Imaging was performed with the Zeiss Axio Imager M2 with 2.5× magnification and 2D-tile recording. Representative images of n=8 slice cultures from 5 animals (1–2 slices per animal). **(B)** Quantification of fluorescent signal intensity of AlamarBlue over time indicating gradual signal reduction on day 10 due to tissue opacity and culture adaption (n=5-12 slice cultures from 4 animals, 2–4 slices per animal). **(C)** HE staining of a brain slice showing preserved tissue architecture and dense cell clusters. Imaging was performed with the Leica DMI3000B at 5× magnification. All data shown as mean ± SD. Statistical analysis was performed using Brown-Forsythe and Welch one-way ANOVA to account for unequal variances between groups using Graphpad Prism 10.

The protocol described herein aimed to generate and establish an *in vitro* GBM model with *ex vivo* components, which recreates GBM growth and behavior *in vitro*. Although a technically demanding task due to the intricate tumor-tissue network and its interactions, our model enables this possibility. To generate a physiologically relevant *ex vivo* GBM model, we established a workflow for the preparation and long-term cultivation of mouse whole-brain slices with invading GBM tumor spheroids (**Figure 1**). In this workflow, neonatal mouse brains (postnatal day 6–9) are embedded in pre-warmed agarose and sectioned into 300 µm thick coronal vibratome slices. Slices are cultured on porous membrane inserts for up to 15 days. By serially sectioning of whole brains (see 3.3), we can produce nine to eleven organotypic slices. Each slice retains the original anatomical organization and cell types. The slices remain viable *ex vivo* and remain their gross morphology without complete submergence in medium. Optimal nutrient supply is achieved by porous membrane inserts which are commonly used for culturing of organotypic slices (15, 26, 29). To ensure that slicing does not negatively affect tissue health, LDS was performed on the day of preparation and repeated over subsequent days to confirm ongoing slice viability (**Figure 2A**). During the initial days (day 0-4) until day 7 whole brain slices retained their compact morphology and remained stable in viability. The fluorescence signal slightly decreased on day 15 following that more reddish areas appear in the center of the slice. Still, the model can be used for extended periods. Slices, which experienced LDS cannot be used for further analysis or staining as it is an end-point-assay. To test viability over time the AlamarBlue assay was performed on equal days. This assay can be repeated on the same slice multiple times (**Figure 2B**). Quantification revealed that there was a statistically non-significant percentage point drop in slice viability or metabolism by 32.8% after day 1, followed by 6 days of stable viability (day 2: 67.2%, day 3: 67.8%, day 4: 69.9%, day 7: 54.1%). After that, a statistically significant change in viability of the whole brain slices to 29.8% (P = 0.0100) was observed (**Figure 2B**). Apart from these fluorescence assays we extended our analyses further to histology. HE staining was performed to test for morphology and anatomy preservation of the brain tissue. It reveals dense cell aggregates embedded within the brain parenchyma, which is surrounded by intact cortical cytoarchitecture (**Figure 2C**). Together, these results establish a reproducible workflow for preparing and maintaining whole brain slice cultures as a platform for studying various diseases including GBM growth, invasion and tumor-brain interactions in native tissue context.

**Figure 3:**
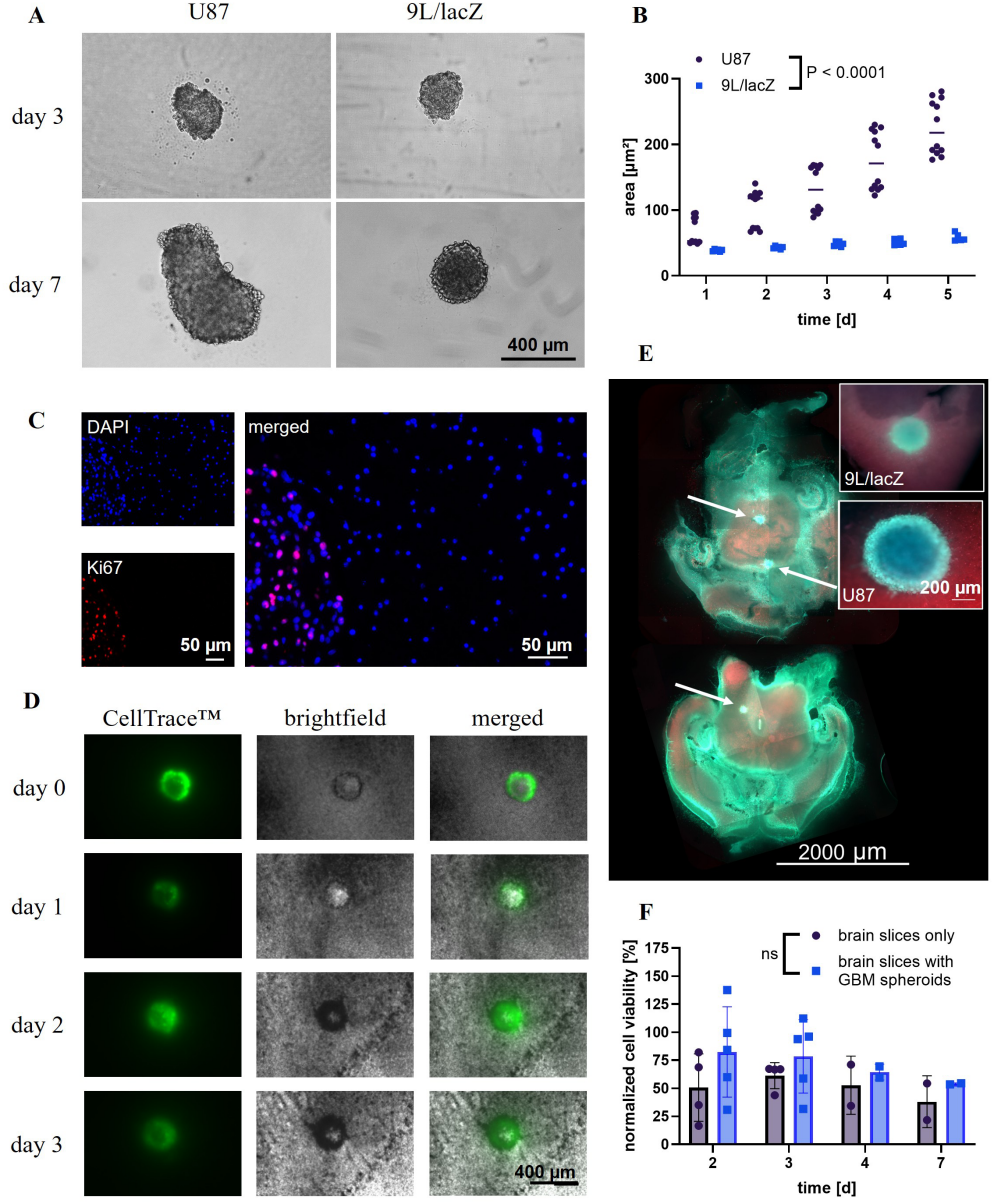
Viability of the 3D GBM model after 96 h co-culture with GBM spheroids. **(A)** Representative brightfield images of U87 and 9L/lacZ GBM spheroids at different time points showing differences in growth rate and morphology (n=12 spheroids in 6 technical and 2 biological replicates). **(B)** Quantification of spheroid area over time obtained from brightfield images using ImageJ (U87: n=8 spheroids in 2 technical and 4 biological replicates; 9L/lacZ: n=6 spheroids in 3 technical and 2 biological replicates). **(C)** Immunofluorescent staining of the proliferation marker Ki67 (red) in U87 cell-spheroids seeded onto whole brain slices after 72 h of culture. Cell nuclei were stained using DAPI (blue). The spheroid is visible on the left side, normal brain slice tissue on the right side of each photograph. Imaging was performed with the Leica DMI3000B at 20× magnification. **(D)** 9L/lacZ-spheroids labeled with CellTrace™ CFSE and cultured on whole-brain slices for 96 h to assess proliferation and tissue integration. Representative fluorescence and overlay images from n=6 slice cultures from 3 animals (2 slices per animal). Imaging was performed with the Leica DMI3000 B with 10× magnification. **(E)** Overview images of whole brain slices displaying decreased tissue viability (red signal) in regions surrounding growing spheroids (arrows) and higher magnification LDS staining (inset) showing that spheroids retain higher metabolic activity compared to surrounding tissue. Representative images from n=8 slice cultures from 4 animals (2 slices per animal). Imaging was performed with the Zeiss Axio Imager M2 with 2.5× magnification and 2D-tile recording (overview) and 10× magnification (inset), respectively. **(F)** Quantification of AlamarBlue fluorescence signal intensities of brain slices co-cultured with and without GBM spheroids over time. All data shown as mean ± SD. Statistical analysis was performed using two-way ANOVA utilizing Graphpad Prism 10.

**Figure 4:**
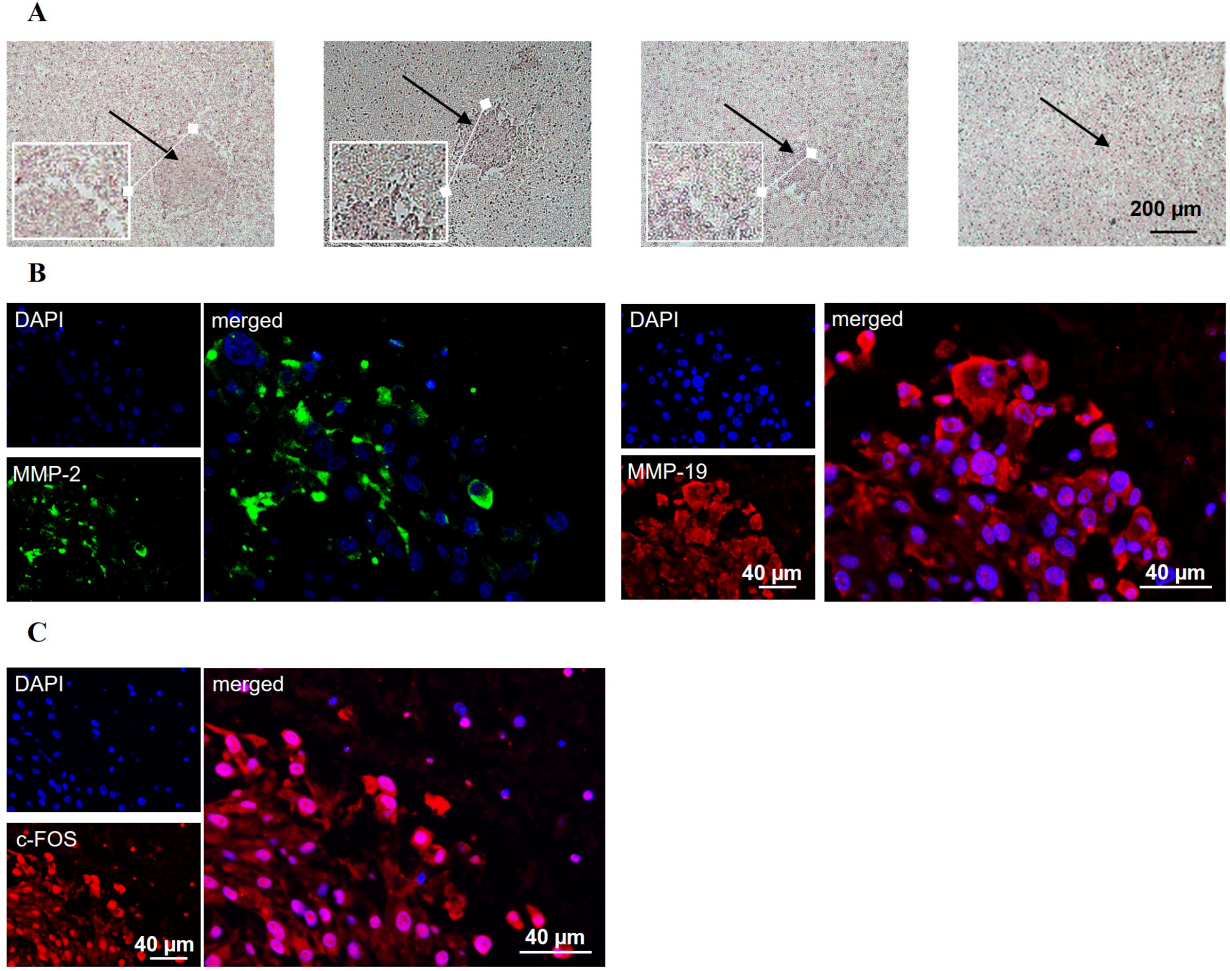
Invasion of U87 GBM-spheroids into the depth of brain slice cultures. **(A)** Hematoxylin-eosin (HE) staining of U87 spheroids after 72 h co-culture showing compact tumor aggregates within the brain slice (arrows) a few µm below the slice surface (left) and in depths of 25 µm, (middle left), 85 µm (middle right) and 100 µm (right). Insets show details of the invasion front. Imaging was performed with the Leica DMI3000B microscope at 5× magnification. **(B)** Immunofluorescence staining of MMP-2 (left, green) and MMP-19 (right, red) combined with DAPI (cell nuclei, blue). Imaging was performed with the Leica DMI3000B microscope at 40× magnification. **(C)** Immunofluorescence staining of c-FOS (red) combined with DAPI (cell nuclei, blue). Imaging was performed with the Leica DMI3000B microscope at 40× magnification.

To test the *ex vivo* organotypic slices for modeling brain tumor growth GBM spheroids of two different cell lines (U87 and 9L/lacZ) were generated in BIOFLOAT^®^ 96-well plates and were then seeded onto the surface of the brain slices. Culturing 500 GBM cells in 100 µL medium produced spheroids of an ideal size for our *ex vivo* grafting experiments (**Figure 3**). By increasing the cell density or spheroid cultivation time, larger spheroids can be formed and employed as a more advanced tumor model. Brightfield imaging of spheroids revealed distinct growth characteristics between the two cell lines. 9L/lacZ spheroids retained a compact and uniform morphology, while U87 spheroids exhibited faster expansion and irregular growth patterns (**Figure 3A**). Quantification of spheroid area confirmed this observation, showing a steady increase in spheroid size from 68.8 µm^2^ on day 3 to 225.9 µm^2^ on day 7 (U87, P < 0.0001) and 38.7 µm^2^ to 58.5 µm^2^ (9L/lacZ, P < 0.0001), respectively, over time (**Figure 3B**). This resulted in a slope of 22.4 µm^2^/d for U87 and 2.8 µm^2^/d for 9L/lacZ cells (P < 0.0001). Immunofluorescence staining of the proliferation marker Ki67 was performed to visualize the proliferative activity of U87-spheroids seeded onto brain slices after 72 h of culture. Within the spheroid, a high proportion of 48% of all cells was positive for Ki67, while there were no Ki67 positive cells detectable in the normal brain tissue of the slice cultures (**Figure 3C**). To verify tumor viability and proliferation within the tissue context, 9L/lacZ spheroids were labeled with CellTrace™ CFSE prior to co-culture. The fluorescent signal remained stable over time, indicating active proliferation and partial invasion into the surrounding brain parenchyma (**Figure 3D**). Whole-slice imaging further showed that spheroid growth was accompanied by a localized reduction in slice viability, visible as red fluorescent regions surrounding the tumor sites, while spheroids appeared more active and vital than the surrounding tissue (**Figure 3E**). However, the general slice viability remained unchanged over 7 days of culture with 82.3% with spheroid vs. 50.6% without on day 2, 78.5% vs. 61.2% on day 3, 64.5% vs. 52.7% on day 4, and 54.0% vs. 38.0% on day 7 (all values not statistically significant) (**Figure 3F**). This suggests that active tumor proliferation affects tissue integrity in the microenvironment in close proximity to the tumor. Hence, cultivation time must be adjusted according to spheroid size and may be limited by the extent of invasion. Histological analysis confirmed the presence of tumor cell clusters within the preserved brain structures a few µm below the slice surface and in depths of 25 µm, 85 µm, and 100 µm after 72 h of co-culture, indicating that the tumor spheroids integrate into the tissue by invasive growth and reach a depth of at least 100 µm (**Figure 4A**). This was accompanied by marked MMP-2 and MMP-19 expression (**Figure 4B**) and high c-FOS detectability within the GBM spheroid, while the normal brain area was negative for MMP expression with single scattered c-FOS positive cells apparent (**Figure 4C**). Overall, these results demonstrate that GBM spheroids remain viable and proliferative within the *ex vivo* brain slice culture, validating the system as a suitable platform for studying tumor-brain interactions under controlled conditions.

## 5 Discussion

Organotypic brain slice cultures originated with Boyd’s pioneering work in 1971, describing a chamber system for large-volume tissue culture (30), were further refined by using roller tube techniques (31) and improved by culturing them on semipermeable membranes (32), which is the standard until today (24). Most subsequent applications utilized regional brain slices, particularly derived from the hippocampus, since they are advantageous in mechanical stability and ease of handling, but sacrifice comprehensive tissue architecture (26, 33). In this study, we present an *ex vivo* whole brain slice model for GBM research, which addresses limitations of region-specific approaches. Spheroids from *in vitro* generated GBM cell lines are grafted to specific brain areas. The tumor spheroids grow or migrate into the brain tissue and induce local measurable changes in the host microenvironment. This *ex vivo* approach bridges the gap between 2D culture systems and animal experimentation by providing a physiologically relevant yet experimentally accessible platform (19). Other recent brain slice models use slices either from adult mice (23) or from 4-week-old male athymic nude mice (34) to investigate tumor cell invasion (19). Slice cultures derived from human surgical tissue have also been described, but are limited due to limited availability and inter-patient variability (19). Coronal slices from rat pups were established for extensive and rapid (4 days) functional drug panel screening by seeding patient tumor tissue or dissociated cell suspensions as multiple tumor foci primarily onto the thalamic regions of each hemisphere (15–18). Our brain slice methodology provides some complementary advantages. We employ whole brain slices from postnatal mice, which are smaller and thereby easier to fit into the vibratome and into the culture-inserts compared to the larger rat brain slices. While the larger rat brains may facilitate technical manipulation and potentially better tumor engraftment, the mouse brains allow better whole-organ preservation due to their smaller size. We are able to generate nine to eleven contiguous slices per brain by leveraging advanced slicing techniques. The slices remain viable up to 15 days and preserve the complete organotypic architecture across the entire organ including the cortex, forebrain, midbrain, cerebrum, and hippocampus. This is of clinical significance, given that the tumor location substantially influences symptoms and patient outcomes (35, 36). It also enables more detailed mechanistic investigations of tumor-brain tissue interactions, allowing to study regional-specific tumor biology and microenvironmental engagement over extended culture periods. This is further supported by utilizing established GBM cell line spheroids, which provide consistency and reproducibility, but of course sacrifice aspects of inter- and intra-tumoral heterogeneity which on the other hand are encountered in patient tumors and of utmost importance for drug screening (15). Therefore, all described methods have the right to exist and complement each other, depending on the research question asked (19). In addition, these models reduce and replace burdensome animal experimentation and are in accordance with the 3R principles (12).

Organotypic brain slices have previously been shown to maintain cytoarchitecture, viability and functional features for extended culture periods (e.g (15, 23, 33, 37, 38).). Our data show that even organotypic slices harvested from virtually the whole brain, remain viable for extended time periods (**Figure 2**), which are long and stable enough for grafting, integration and growth of tumor spheroids from different origin (**Figure 3**). The ability to monitor tumor-brain interactions over time is critical, given that invasive growth and microenvironment engagement are clinically relevant hallmark features of GBM (21, 23, 39).

Our observation that GBM spheroids influence local slice viability (**Figure 3**) suggests that the model captures not only tumor growth but also host tissue responses. This is consistent with prior work showing that tumor cells in brain slice models can induce changes in local tissue compartments, including gliosis, microglial activation or extracellular matrix remodeling (37, 40). The co-culture of GBM with brain slices thus provides an improved microenvironmental context compared to spheroids alone in suspension. Moreover, the differences we observed between U87 and 9L/lacZ spheroids (**Figure 3**) highlight that the model can reflect cell line specific growth behavior and perhaps pathophysiological heterogeneity. The human-derived U87 cells are widely used in GBM-research, especially since they exhibit significant proliferative capacity and invasive properties (41). Subpopulations of these cells have been shown to display variable invasion and proliferation characteristics (41), which are strongly regulated by microenvironmental factors. These comprise substrate rigidity (42), hypoxic conditions (43), and glucose availability (44). The invasion capacity of these cells is mediated by increased expression of several metalloproteinases, particularly MMP-2, MMP-9, and MMP-19 (45, 46). 9L/lacZ cells are nitrosourea-induced gliosarcoma cells with an aggressive and invasive phenotype displaying high tumorigenicity *in vivo* (47). These cells are widely used in preclinical glioblastoma research (48). Compared to monolayer cell cultures, they display aggressive cellular behavior, including enhanced migration and invasion, when grown as 3D-spheres (49). 9L cells have been shown to invade surrounding brain parenchyma involving matrix metalloproteinases (50) and to display pronounced proliferation with doubling times of 2.4-2.6 days *in vivo* (51). Indeed, MMP-2 and MMP-19 expression were detectable in the tumor spheroids invading our brain slices (**Figure 4**). c-FOS has been described as a marker for neuronal activation (52) and such activation is functionally linked to GBM invasion (53–55). We observe c-FOS expression in the GBM spheroids as well as positive cells scattered within the normal brain area of the whole brain slice cultures (**Figure 4**). Thus, both these cell lines are well suited to investigate diverse facets of GBM aggressiveness, including proliferation and invasion utilizing our whole brain slice model.

Brain slices from different regions - including hippocampal (33), coronal (56), and cerebellar (57) compartments - can be prepared from both wild-type and transgenic animals. Notably, only about 5% of GBM tumors occur in the cerebellum, and tumor location significantly influences symptoms and clinical outcomes (2). Therefore, it is crucial to develop a comprehensive GBM brain slice model that encompasses the entire organ rather than focusing on isolated regions.

Our model offers several advantages: (i) the preservation of native brain tissue architecture and multiple cellular components, (ii) a reduction in animal usage by generating multiple slices per brain, (iii) compatibility with live imaging, quantitative read-outs and histological validation (29). Methods-based studies such as ours benefit from these features when aiming to establish robust proof-of-concept systems. Some limitations merit discussion (29). Although the slices preserve many aspects of the brain microenvironment, they lack vascular perfusion, systemic immune components and long-term organism-level homoeostasis. Hence, our model cannot completely replace *in vivo* models for studies investigating brain tumor features like migration over the blood-brain-barrier, systemic pharmacokinetics or immune-system interactions (29). Second, the depth of tumor invasion into the slice may be limited compared to *in vivo* infiltration. Several reports show that while organotypic slice models permit investigation of migration/invasion, they may not fully recapitulate the complete fractional depth or axis of invasion seen in patients (21, 23, 29, 58). Third, slice culture viability, especially in older brains or extended culture durations, may decline, potentially confounding long-term studies (29, 59). Our data show viability up to 15 days, a time frame comparable to many published systems (37, 39). However, longer-term culture beyond 2 weeks may require further optimization or validation.

Looking forward, the model can be applied in multiple directions: (i) assessing tumor-microenvironment interactions under treatment (e.g. chemotherapy, radiation, novel therapeutics) in a near-native tissue setting, (ii) comparative studies of multiple GBM lines and patient-derived organoids to evaluate heterogeneity of growth/invasion/behavior, (iii) mechanistic interrogation of cell-brain interactions (e.g. ECM remodeling, astrocyte/tumor cell cross-talk, microglial activation) in a controlled *ex vivo* setting, (iv) screening of therapeutic interventions in a medium-throughput format, thereby reducing reliance on large-scale *in vivo* experiments (15, 18).

In conclusion, the workflow described herein provides a practical, biologically relevant and experimentally amenable *ex vivo* brain slice model for GBM spheroid co-culture. It supports tumor viability, growth and microenvironmental interaction for up to 15 days, and offers a versatile platform for mechanistic and pre-clinical investigations. We anticipate that the adoption of such models will contribute to bridging the translational gap between *in vitro* simplifications and *in vivo* complexity, facilitating more predictive studies of tumor progression and therapy in GBM.

## Data Availability

The raw data supporting the conclusions of this article will be made available by the authors, without undue reservation.
